# A Cross-sectional Study of QOL of Diabetic Patients at Tertiary Care Hospitals in Delhi

**DOI:** 10.4103/0970-0218.58397

**Published:** 2009-10

**Authors:** Yogesh Gautam, AK Sharma, AK Agarwal, MK Bhatnagar, Roochika Ranjan Trehan

**Affiliations:** Department of Community Medicine, SJH & VMMC, New Delhi - 110 029; 1 Lady Hardinge Medical College, New Delhi- 110 001; 2 Dr RML Hospital, New Delhi- 110 001; 3 LHMC & Assoc. SSKH, New Delhi- 110 001; 4 LHMC & Assoc. SSKH, Department of Medicine, New Delhi- 110 001

**Keywords:** Diabetes, QOL, SF-36

## Abstract

**Background:**

According to WHO estimates India will be the global capital of diabetes by 2025, accounting for 57.2 million diabetics. Worsening the situation is the fact that diabetes affects the economically productive age-group (45-65 years) in developing countries.

**Objective:**

To measure quality of life (QOL) and study the clinical profiles and associated sociodemographic factors affecting diabetic patients aged 20 years and above.

**Materials and Methods:**

We conducted a hospital-based cross-sectional study using a generic instrument, Short-Form 36 (SF-36 of the Medical Outcome Study Group) to measure QOL of diabetic subjects aged ≥20 years. Two hundred and sixty diabetics, including 91 males and 169 females, were selected from the clinics of SSK Hospital and Dr RML Hospital of New Delhi. Data was analysed using SPSS for Windows, version 12.

**Results:**

The mean age of the respondents was 49.7 years, with 80% of respondents being in the age-group of 40-69 years. The majority (52.1%) of female respondents were illiterate and 91.1% were economically dependent. Of the male respondents, 65.9% were skilled workers. Substance abuse was present among 41.8% male subjects. Type 2 diabetes was the commonest, with 94.6% of the subjects having this form. The mean duration of diabetes was 6.96 ± 6.08 years. Oral hypoglycemic agents were being taken by 70.77% of the respondents. Among the diabetics the most common comorbidity was hypertension (30.8%) and the commonest complication was neuropathy (26.2%). We calculated the body mass index (BMI) of all subjects and found that, 46.2% of the male and 59.8% of the female respondents were either overweight or obese. As predicted by the waist/hip ratio (WHR), 53.8% of the male and 66.9% of the female respondents had high risk for CHD. Regular physical activity was undertaken by less than half of the subjects (46.5%). Out of eight domains of QOL in the SF-36, the two most affected were ‘General Health’ and ‘Vitality.’ Overall, males had higher QOL scores; this was found to be statistically significant (*P* = 0.0001). SF-36 and its eight domain scores had significant association with socioeconomic status, education, and habitual physical activity.

**Conclusion:**

Diabetes had an adverse effect on the QOL of these study subjects. Females had a significantly poorer QOL than males. The domains most affected were ‘General Health’ and ‘Vitality.’ Poor scores in the QOL domains were significantly associated with lower socioeconomic status, lesser education, and lesser habitual physical activity.

## Introduction

Diabetes mellitus has reached epidemic proportions. According to the World Health Organization (WHO) there is *‘an apparent epidemic of diabetes, which is strongly related to lifestyle and economic change.’* India has the largest number of diabetics in the world. There were around 19.3 million diabetics in India in 1995 and the projected figure for the year 2025 is 57.2 million.(1)

Diabetes is a chronic disease related to lifestyle. It has a negative impact on the affected individual's perception of wellbeing. ‘Wellbeing’ is presently considered difficult to measure because of the subjective nature of perception and responses. In the present study we attempted to measure the quality of life (QOL) of diabetics through a personal interview and the SF-36 questionnaire. Proper drug therapy, social support, health education, and psychological care in diabetes are essential but are usually deficient, especially in developing countries.

QOL is a holistic concept which addresses many aspects of health. It has been defined by WHO as ‘*an individual's perception of their position in life in the context of culture and value system in which they live and relation to their goals, expectations, standards, and concerns*.’(1) In this context the present study addresses the issue of QOL among diabetics in Delhi.

This study is expected to increase the knowledge of the impact of diabetes on patients' lives, i.e., their physical, mental, and social wellbeing. With chronic diseases claiming more lives than other types of diseases, effective strategies need to be developed and action plans formulated to help the affected.

## Materials and Methods

### Study area

We carried out this hospital-based cross-sectional study among the patients attending the diabetic clinics of hospitals associated with Lady Hardinge Medical College (LHMC), i.e., Smt. Sucheta Kriplani Hospital (SSKH) and Dr Ram Manohar Lohia Hospital (RMLH), New Delhi. These two hospitals are situated barely a kilometre apart and Therefore the population served by these two hospitals is from the same catchment area. The study was carried out from January 2006 to December 2006.

### Study unit

Patients aged 20 years and above, and on treatment for diabetes for at least 3 months, were included in the study. Patients having gestational diabetes and major psychiatric disorders were excluded from the study as these have been identified as potential confounding factors.

### Sampling unit

Patients registered on the day of interview were selected using systematic random sampling. On an average, 3–5 respondents were interviewed per clinic per day; clinics were held 2 days a week.

### Sample size

Two hundred and sixty subjects were finally included in the study (calculated through convenience sampling were 240 i.e. 40 weeks × 2 clinic days × 3 subjects = 240).

### Study instrument

A predesigned, pretested semistructured interview schedule was used. Informed consent was taken for interviewing subjects. The prospects of this study for improving understanding of diabetes was explained to the participants. The response rate was 97%. At interview, we collected data on personal details, treatment history, and relevant clinical history. This was followed by a general physical and systemic examination. A standardized questionnaire, viz the MOS SF-36 v2 (Hindi version), was used to measure QOL of diabetic patients. This questionnaire has eight domains, viz Physical Functioning (PF), Role Physical (RP), Bodily Pain (BP), General Health (GH), Vitality (VT), Social Functioning (SF), Role Emotional (RE), and Mental Health (MH). These domains were scored on a scale of 0–100, ‘0’ indicating the worst possible status and ‘100’ the best possible status. The scoring manual of *Ware et al.*(11) was used for calculating scores. Data entry was done in SPSS, version 12, followed by reverse coding of 10 items. Thereafter, raw scale scores were deduced and were finally transformed to a scale of 0–100.

## Result

Out of the 260 respondents, 169 were females and 91 were males. The mean age of the respondents was 49.7 years (M = 48.8 years; F = 50.1 years). Eighty percent of the respondents were in the age-group of 40–69 years. The majority (59.6%) were from the middle socioeconomic status (SES); 35.0% belonged to the lower SES according to the modified Kuppuswamy's socioeconomic status scale [as per CPI (consumer price index) 2003].(10) Of the 260 respondents, 75.0% were married; 63.8% belonged to nuclear families, and 36.1% belonged to joint families. The majority (52.1%) of female respondents were illiterate, but only 11.0% of male respondents were illiterate. The majority (65.9%) of male respondents were skilled workers and 83.6% of the females were homemakers. Most (91.1%) of the female respondents were economically dependent, while only 25.3% of the males were economically dependent. Substance abuse (alcohol and tobacco) was present among 41.8% of male and 8.9% of female respondents.

The majority (94.6%) of the study subjects had type 2 diabetes. The mean duration of diabetes was 6.96 ± 6.08 (SD) years. The majority (70.77%) of respondents were on oral hypoglycemic agents. The most (30.8%) common comorbidity was hypertension. The most common complication present was neuropathy (26.2%). The most common dental morbidity among study subjects was dental caries (29.6%).

A large proportion of the male (46.2%) and female (59.8%) respondents were either overweight or obese. Regular physical activity was undertaken by less than half of the subjects (46.5%). A waist/hip ratio (WHR) of more than 0.95 in males and 0.85 in females is considered as indicating risk for coronary heart disease (CHD). By this criterion, 53.8% of the male and 66.9% of the female respondents had high risk for CHD [Table 1].

**Table 1:** Clinical profile of study subjects

Clinical profile Variables	Male (*n* = 91)	Female (*n* = 169)
Nutritional status (as per BMI)		
Normal	46.2%	36.7%
Overweight and obese	46.2%	59.8%
Underweight	7.7%	3.5%
Waist/Hip ratio (WHR)		
No risk		
(< 0.95 M; <0.85 F)	46.2%	33.1%
Risk present		
(> 0.95 M; >0.85 F)	53.8%	66.9%
Duration of diabetes (mean duration 6.96 ± 6.08 years)		
<1 year	8.7%	10.8%
1–5 year	38.7%	38.5%
5–10 year	27.5%	28.3%
>10 year	25.1%	22.3%
Comorbidity*		
Absent	60.4%	43.8%
Present	39.6%	56.2%

* Multiple comorbidities were present in 7.7% of males and in 14.8% of females.

Hypertension was the most common comorbidity, being present in 30.8% and 45.6% of the males and females, respectively

QOL was assessed using the MOS SF-36 questionnaire (v2). Self-appraisal of current health status was better among male (91.2%) than among female (76.3%) respondents. When asked to assess their health status as compared to that experienced 1 year back, both male (63.8%) and female (57.5%) subjects reported that it had worsened or that there had been no change in health status. Out of eight domains in the SF-36 questionnaire, the two most affected domains were GH and VT. The two domains that were least affected were SF and RE. Overall, males had higher QOL scores as compared to females [Table 2]. This difference was found to be statistically significant (*P* = 0.0001).

**Table 2:** Distribution of SF-36 scores by sex of study subjects

Sf-36*	Sex		Domain score
		
	Male	Female	
Physical functioning	78.68 ± 25.46	58.37 ± 26.15	65.48 ± 27.63
Role physical	71.22 ± 27.84	51.85 ± 26.81	58.63 ± 28.66
Role emotional	77.65 ± 26.60	63.22 ± 28.67	68.27 ± 28.64
Bodily pain	69.63 ± 23.46	54.78 ± 21.65	59.98 ± 23.36
General health	54.23 ± 16.95	42.54 ± 13.17	46.63 ± 15.60
Vitality	58.38 ± 19.81	43.93 ± 18.16	48.99 ± 19.95
Social functioning	79.94 ± 22.81	63.16 ± 25.30	69.04 ± 25.70
Mental health	65.82 ± 15.40	54.88 ± 18.68	58.71 ± 18.33
Sf-36 score	69.44 ± 16.68	54.09 ± 17.53	59.47 ± 18.70

* SF-36 scores from 0–100 (**0** represents worst & **100** best quality of life); {ANOVA *P* = 0.0001 significant for all domains

When the SF-36 scores and its sub-domains were compared against various sociodemographic and clinical parameters, significant associations were observed [Table 3]. The SF-36 and its eight domains scores were found to have statistically significant association with socioeconomic status, education, and habitual physical activity. All domains, other than GH, had significant association with age and marital status.

**Table 3:** Comparison of SF-36 scores and salient characteristics of subjects

Variable	Sub classification	Domains								SF36
			
		PF	RP	RE	BP	GH	VT	SF	MH	
Age groups	20–39, 40–59, >60	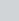	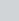	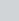	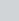	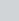	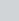	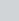	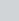	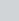
SES	UC, UMC, LMC, ULC, LC	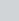	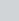	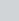	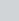	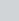	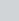	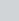	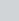	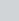
Duration	0–2, 2–5, 5–10, >10 yr		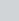	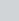	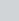		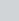		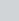	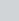
Family type	Nuclear, joint									
Education		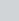	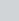	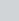	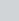	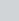	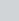	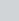	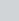	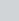
Marital Status	Married, widow, single	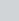	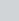	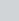	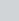		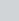	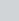	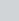	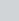
BMI	Malnourish, normal				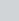	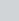				
Treatment	OHA, insulin									
Glycemia	Normo- and hyperglycemia	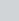	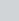	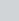	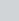	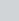		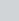		
HPA*	(regular, no/irr.)	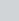	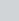	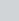	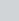	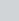	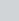	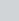	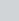	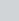
Complication	Present, absent	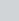	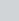	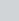						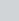
Comorbidity	Present, absent	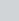								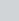

* HPA = Habitual physical activity. 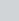 Shaded boxes represents statistically significant relation, i.e., *P* ≤ 0.05

## Discussion

In the present study, we interviewed 260 subjects, most of whom were urban residents. The uneven sex distribution among the study subjects is due to administrative differences. The diabetic clinic of Dr RMLH caters to CGHS (Central Government Health Service) scheme beneficiaries, while the clients of SSKH are mainly postmenopausal women, most of whom have less social support. Type 2 diabetes was present in 94.6% of subjects, which is consistent with the observed prevalence of type 2 diabetes worldwide.(1) Eighty percent of the respondents were in the age-group of 40–70 years, which is consistent with the pattern of diabetes observed in developing countries.(1) The mean weight of the respondents was 62.90 ± 13.13 kg (males: 65.90 ± 12.91 kg; female: 61.28 ± 13.09 kg). The mean body mass index (BMI) of males was 25.03 ± 4.64 kg/m^2^ and of females 27.01 ± 5.48 kg/m^2^, while the mean WHR was 0.95 ± 0.10 and 0.88 ± 0.09 among males and females, respectively. The mean duration of diabetes among respondents in the present status was 6.96 ± 6.08 years. In comparison, Okanovic(3) (Croatia) and Subratty(2) (Mauritius) have reported a mean duration of diabetes in their study subjects of 10.2 ± 6.2 years and 9.3 ± 7.7 years, respectively. The most common complication observed in our diabetic patients was neuropathy (26.2%), which is consistent with the findings reported by other authors; for example, Jacobson *et al*.(4) reported that 48.8% of their subjects had neuropathy; Mayou and Bryant(5) found 20% with neuropathy; and Weinberger *et al.*(7) reported 24% with neuropathy.

Overall, the SF-36 score was lower (54.09) in females than in males (69.44) and this difference was statistically significant (*P* = 0.0001). Males had higher scores than females in all eight domains. Chittleborough *et al.*(7) reported similar findings in an Australian population, where the QOL scores among males were higher in all domains, except in GH and VT. Gulliford and Mahabir(8) have reported that SF-36 scores, as also individual domain scores (except RP and RE) were more in males in Trinidad and Tobago. Woodcock *et al*.(9) in the UK reported better scores for males in all domains, except in BP. As regards to QOL in those with duration of diabetes of more than 5 years, we found that these subjects had lower scores in all domains except in GH and MH, which may be due to improvement in GH and MH domains due to adaptation to diabetic lifestyle, while rest has opposite effect. Woodcock *et al.*(9) also noted that subjects with more than 5 years' duration of diabetes had better scores in all domains, except in BP. Overall, the SF-36 score was significantly lower among respondents with complications as compared to respondents with no complication; PF, RP, and RE were affected more and the differences were statistically significant. Woodcock *et al*.(9) also observed better scores in all domains (except RP and BP) in those without complications.

### Limitations and bias

1. Hospital-based study
2. The findings cannot be generalized, as they may not be applicable to diabetics with duration of diabetes of more than 5 years
3. A comparison group of non-diabetic subjects was not included in study

## Conclusions

The mean age at diagnosis of diabetes was found to be 50 years. Thirty-five percent of the subjects were from the lower SES, which is confirmation that the epidemic of diabetes is not limited to the upper classes.(1) The majority (63.8%) of the diabetics are from nuclear families, indicating that they do not have the special support that is available in the traditional Indian joint family. Poor socioeconomic status and low literacy rates in the female subjects prevent them from receiving the care needed to achieve adequate QOL. Forty-two percent of the male subjects reported substance abuse (alcohol and/or tobacco). QOL is affected significantly by key factors like SES, literacy, and habitual physical activity. As duration of diabetes increases, patients feel better as they come to terms with their diabetic condition, however physical health deteriorates. Males adapt better to life with diabetes as compared to females. Married patients reported better QOL.

## Recommendations

We suggest all of the following for improving QOL in diabetes: regular meditation, creating self-help groups, physical activity of a minimum 30 min/day, good glycemia control, improving female literacy, improving social support system, avoidance of substance abuse, and making counselling services available. Further studies of data of different disease assessed by SF-36 can contribute to the understanding of the relative impact on QOL of various diseases.
